# Invasion History of the Pinewood Nematode *Bursaphelenchus xylophilus* Influences the Abundance of *Serratia* sp. in Pupal Chambers and Tracheae of Insect-Vector *Monochamus alternatus*

**DOI:** 10.3389/fpls.2022.856841

**Published:** 2022-05-20

**Authors:** Haokai Tian, Tuuli-Marjaana Koski, Lilin Zhao, Ziying Liu, Jianghua Sun

**Affiliations:** ^1^State Key Laboratory of Integrated Management of Pest Insects and Rodents, Institute of Zoology, Chinese Academy of Sciences, Beijing, China; ^2^CAS Center for Excellence in Biotic Interactions, University of Chinese Academy of Sciences, Beijing, China; ^3^College of Life Science, Institute of Life Science and Green Development, Hebei University, Baoding, China

**Keywords:** *Serratia marcescens*, pinewood nematode, *Monochamus alternatus*, pathogenicity, bacteria isolation, invasion time

## Abstract

Pine wilt disease (PWD) has caused extensive mortality in pine forests worldwide. This disease is a result of a multi-species interaction among an invasive pinewood nematode (PWN) *Bursaphelenchus xylophilus*, its vector *Monochamus* sp. beetle, and the host pine tree (*Pinus* sp.). In other systems, microbes have been shown to attenuate negative impacts on invasive species after the invasion has reached a certain time point. Despite that the role of PWD associated microbes involved in the PWD system has been widely studied, it is not known whether similar antagonistic “hidden microbial players” exist in this system due to the lack of knowledge about the potential temporal changes in the composition of associated microbiota. In this study, we investigated the bacteria-to-fungi ratio and isolated culturable bacterial isolates from pupal chambers and vector beetle tracheae across five sampling sites in China differing in the duration of PWN invasion. We also tested the pathogenicity of two candidate bacteria strains against the PWN-vector beetle complex. A total of 118 bacterial species belonging to 4 phyla, 30 families, and 54 genera were classified based on 16S sequencing. The relative abundance of the genus *Serratia* was lower in pupal chambers and tracheae in newly PWN invaded sites (<10 years) compared to the sites that had been invaded for more than 20 years. *Serratia marcescens* strain AHPC29 was widely distributed across all sites and showed nematicidal activity against PWN. The insecticidal activity of this strain was dependent on the life stage of the vector beetle *Monochamus alternatus*: no insecticidal activity was observed against final-instar larvae, whereas *S. marcescens* was highly virulent against pupae. Our findings improved the understanding of the temporal variation in the microbial community associated with the PWN-vector beetle complex and the progress of PWD and can therefore facilitate the development of biological control agents against PWN and its vector beetle.

## Introduction

The pinewood nematode (PWN), *Bursaphelenchus xylophilus* (Steiner and Buhrer) Nickle, is native to North America and is the causal agent of a highly destructive pine wilt disease (PWD). This nematode has become an invasive global quarantine pest and has damaged pine forests in Japan, South Korea, China, and recently also in Portugal and Spain (Mota et al., 1999; Zhao et al., 2014). In China, despite tremendous prevention and control efforts implemented during the past 40 years, PWN has recently spread from its initial southern distribution range to the high-altitude range in the northern part of the country (Zhao et al., 2014; Pan et al., 2020). Compared to many other plant-parasitic nematodes, PWN has a complex life cycle including both intermediate vector (*Monochamus* sp. beetle) and primary (*Pinus* sp.) hosts, to which PWN life stages are tightly connected. Propagative stages (egg to adult) live exclusively within the host pine whereas the dispersal life stages (J_III_ and J_IV_) are also associated with the vector beetle. Under unfavorable conditions, propagative stages molt to third-stage dispersal juveniles (J_III_) and aggregate around the pupal chamber to wait for beetle pupation. Once the vector beetle has reached the late pupal stage or early adulthood, J_III_ molts to fourth-stage dispersal juveniles (J_IV_) and enters the vector beetle's tracheae system. After the vector beetles mature and emerge from the PWN invaded trees, they find suitable trees to feed and oviposit and transport J_IV_ in their tracheae to new pine trees where the nematodes start a new life cycle (Zhao et al., 2013b, 2016). Besides needing to balance the different environmental conditions generated by the host tree and insect vector, PWN also encounters various microbiota in both environments, which may influence their invasion success. For example, bacteria and fungi associated with PWD can influence nematode reproduction and virulence, the population size and prevalence of the PWN-vector beetle complex, the severity of PWD, and the resistance of host pine (Vicente et al., 2012; Zhao et al., 2013a; Nascimento et al., 2015; Alves et al., 2018; Zhang et al., 2020b).

After the successful colonization, the population size of an invasive species usually increases but may eventually start to decline after the invasion has reached a certain time point (Knevel et al., 2004; Nijjer et al., 2007; Diez et al., 2010; Zhao et al., 2019). This decline may be related to the biological resistance formed by the native biotic and abiotic factors (Mitchell et al., 2010). One of such factors can be an antagonistic microorganism associated with the invasive species. Past studies have shown that the initial dominance of an invasive species can later be reversed by stabilizing processes, which in some cases are mediated by changes in associated microbiota. For example, the biomass accumulation of invasive plants in the invaded habitat can decrease due to an increase in the abundance of local soil pathogens (Nijjer et al., 2007; Diez et al., 2010; Mitchell et al., 2010). In the PWD system, only a few studies have investigated the changes in the microbial community during the progress of PWD, leaving the temporal effects on microbiota composition unknown. However, pupal chambers in sites with a longer duration of PWN invasion significantly harbor fewer nematodes compared to sites with a shorter duration of invasion (data not shown). Because PWN is also in contact with various microbial associates, it is possible that similar to the example from some invasive plants, the increased proportion of antagonistic microorganisms may also explain the lower abundance of PWN in these sites.

Pinewood nematodes and vector beetles are known to be associated with several fungi, many of which are shown to be beneficial for this disease complex. Some fungi such as *Ophiostoma ips, Ophiostoma minus*, and *Ceratocystis* sp. act as a nutritional source for the mycophagous phases of PWN (Vicente et al., 2021). Ophiostomatoid fungi (i.e., *Sporothrix* sp. 1), dominating in pupal chambers and vector beetle tracheae, also positively influence the reproduction of PWN and increase the growth and survival rate of its vector beetle (Futai, 2013; Zhao et al., 2013a). Regarding PWN associated bacteria, the genus *Serratia* widely exits across all stages of PWN's life cycle (i.e., both propagative and dispersal stages) and appears to be a close associate of PWN, having increased relative abundance in pines with PWD (Guo et al., 2020; Zhang et al., 2020b). Some *Serratia* species help PWN to survive from high oxidative stress (Vicente et al., 2013), whereas some species of this genus are potentially harmful to PWN while being beneficial to the host plant (Paiva et al., 2013; Akduman et al., 2018; Liu et al., 2019). *Serratia plymuthica* M24T3, for instance, is nematicidal but its presence can improve the tolerance and the growth of its host plant, *Arabidopsis thaliana* (Proenca et al., 2019). Similarly, *Serratia quinivorans* BXF1 is antagonistic to fungi and bacteria but promotes the growth of plant roots and can also opportunistically colonize the same host pine species together with PWN (Nascimento et al., 2016). Besides being potentially antagonistic to PWN, some *Serratia* species may also be harmful to PWN's vector beetle (*Monochamus* sp.), because bacteria of this genus are conditionally pathogenic to more than 70 species of insects (Grimont and Grimont, 1978). For example, *Serratia marcescens* is lethal if it enters the hemolymph and can successfully evade the immune system of the insect (Raymann et al., 2017, 2018). Interestingly, some vector beetle adults and larvae (e.g., *M. alternatus, M. galloprovincialis*, and *M. saltuarius*) that have a phoretic relationship with PWN can also carry genus *Serratia* in their gut microbiota (Vicente et al., 2013; Alves et al., 2016; Guo et al., 2020; Ge et al., 2021) without adverse effects under normal conditions.

So far, no research has focused on PWN associate bacteria-to-fungi ratio or investigated the temporal changes in their abundances as the PWN invasion progresses. Despite the positive effects of the dominating Ophiostomatoid fungal associates, we speculated that bacteria unfavorable to the PWN-vector beetle complex may be the “hidden players” behind the observed lower PWN abundance in areas with a longer duration of PWN invasion. More specifically, we hypothesized that some bacteria (i.e., *Serratia* sp.) pathogenic to PWN and its vector beetle will increase in abundance as the invasion progresses, being more abundant in the sites with a longer duration of PWN invasion. We tested this hypothesis by investigating the microbiota community in pupal chambers and vector beetle tracheae in sites differing in the duration of PWN invasion, as well as using candidate bacterial isolates from these samples to investigate their pathogenicity to PWN and its vector beetle. We focused on the genus *Serratia* because of the frequent association of these bacteria with PWD as well as the high potential of these bacteria to negatively influence both PWN and its vector, therefore being excellent biocontrol candidates against PWD.

## Materials and Methods

All samples in this study were collected from five locations in China in the spring for 2 years (March to April 2018–2019). These five sites varied in the duration of PWN invasion: Liaoning (LN): 1–5 years of invasion; Shaanxi (SX) and Anhui (AH): 5–10 years of invasion; Zhejiang (ZJ): 15–20 years of invasion; and Jiangsu (JS): 25–30 years of invasion (Supplementary Figure 1). Thirty trees with signs of late-stage PWD infection (recently dead and leafless) with a diameter at breast height (DBH) of 10–15 cm were sampled per location. Two or three logs of 20–30 cm long and of 5–10 cm in diameter were chosen from each tree (Zhao et al., 2013a). The pine species in each location were LN: *Pinus tabuliformis, Pinus koraiensis*; SX: *Pinus tabuliformis* and *Pinus armandi*; AH, ZJ, and JS: *Pinus massoniana* (Supplementary Figure 1). The logs from each site were kept at 4°C until used for pupal chamber samples and J_III_ collection (2018–2020). The rest of the logs were kept in the glasshouse for vector beetle collection (2018–2019).

### Collection of Pupal Chamber Samples

Each year, pupal chamber samples of *Monochamus* sp. vector beetles were collected from the randomly chosen logs from each site. Sawdust was scraped using a sterile scalpel and forceps from the surface of pupal chambers across multiple logs, and tissues from three to five pupal chambers were pooled as one sample. In total, five to seven samples were collected from each site per year, resulting in a total of 87 samples from the five sites across the two sampling years (2018 and 2019).

### Collection of Vector Beetle Tracheae Samples

The vector beetles (*M. alternatus* and *M. saltuarius*) were surface-sterilized by using ethanol and then rinsed three times with sterile water. Tracheae of vector beetles were extracted under the CKX53 microscope (Olympus, Tokyo, Japan) (Zhou et al., 2018; Zhang et al., 2020a). In total, we collected 394 vector beetles. From each beetle, we extracted one trachea sample including all tracheal tissue, resulting in a minimum of 30 tracheae samples from each site per year.

### Nematode Collection

J_III_s were collected in the tissues surrounding pupal chambers from the same logs in which pupal chambers were sampled. In brief, 20 g of wood tissue around each pupal chamber was used to recover PWN by the using Baermann funnel technique (Zhao et al., 2013a,b). After collection, J_III_ was examined under a microscope for morphological confirmation.

### Fecundity of Nematodes Collected From Sites Varying in the Duration of PWN Invasion

The J_III_s were cultured with the fungus *Botrytis cinerea* on potato dextrose agar (PDA) plates in which they can molt to propagative stages. Periodic investigation of nematode fecundity was made by choosing 12 individuals (nine females and three males) of PWNs to a PCR tube containing 150 μl of phosphate-buffered saline with tween-20 (PBST). Each treatment was repeated 10 times, the total number of eggs at the bottom of each tube was counted under a microscope (Meng et al., 2020). To confirm whether the potential differences in fecundity were related to the differences in the duration of PWN invasion, we compared the fecundity (mean number of eggs) of PWN harvested from different sites after these nematode populations were cultured in the lab for 7 days and again after 30 days of culturing.

### Quantification of Relative Bacterial and Fungal Abundances

The DNA extraction of the pupal chamber and tracheae samples was performed using the High Pure PCR Template Preparation Kit (Roche Applied Science, Mannheim, Germany) following the manufacturer's procedures. The DNA samples extracted from pupal chambers and tracheae used for Illumina sequencing for microbiota investigation were also used for the PCR analysis of bacterial and fungal abundance. According to previous studies, the bacterial genus *Serratia* and the fungal genus *Sporothrix* are widespread and dominant taxa in the pupal chambers (Zhao et al., 2013a; Guo et al., 2020), which is why these taxa were used for plasmid synthesis. For the bacterial standard, a 203-bp, the double-stranded plasmid was synthesized to span the 16S region of the *S. marcescens* genome. For the fungal standard, a 333-bp, double-stranded DNA plasmid was synthesized to span the 18S internal transcribed spacer (ITS) region and the 5.8S rRNA gene of *Sporothrix* sp. 1. The standard recombinant plasmids of two strains were performed using *pEASY*-T5 Zero Cloning Kit according to the manufacturer's instructions (Transgene, Beijing, China). For each taxon's standard, the lyophilized material was used to prepare a 10 nM stock solution that was serially diluted to produce 10 standards spanning the 10^−1^-10^8^ copy number range (Ledon-Rettig et al., 2018; Tkacz et al., 2018). Plots of Cp vs. log 10 (copy number) afforded linear calibration curves with amplification efficiencies between 91–95% and 72–73% for *S. marcescens* and *Sporothrix* sp.1, respectively, and *R*^2^ values of 1 and 0.96–0.98 for these taxon standards, respectively. Based on the melting-curve analyses, we found no evidence for primer dimers. We estimated fungal and bacterial abundance based on the estimated gene copy number from their respective standard curves and used these values to calculate bacteria-to-fungi ratios (Fierer et al., 2005).

The samples used for microbial profiling were used for RT-qPCR (five biological replicates). The RT-qPCR annealing temperature was 49°C and the extension time was 45 s. A fragment of the 16S ribosomal ribonucleic acid (rRNA) gene was amplified using the primers Eub338 (5′-ACTCCTACGGGAGGCAGCAG-3′) and Eub518 (5′-ATTACCGCGGCTGCTGG-3′) for bacteria and a fragment spanning the SSU internal transcribed spacer (ITS) region and the 5.8S rRNA gene for fungi using primers ITS1f (5′-TCCGTAGGTGAACCTGCGG-3′) and 5.8S (5′-CGCTGCGTTCTTCATCG-3′) (Fierer et al., 2005).

### Analysis of Relative Abundance of the Genus *Serratia* Among Different Sites

The microbiota data from the pupal chamber and vector beetle tracheae samples used in this study have been submitted to the NCBI BioProject under accession number PRJNA720535. The relative abundance of genus *Serratia* involved in pupal chambers and tracheae of vector beetles have based on the ASV table of our other experiment (Tian et al. unpublished data). The ASV tables used for analyses in this study are available at https://github.com/Haokai-Tian/Tian2022_microbiome.git, but the results are presented here (Figure 4) for a demonstration of the microbial community differences among the sites.

### Isolation and Identification of Bacteria From the Pupal Chamber and Tracheae Samples

Pupal chamber and beetle tracheae samples were surface-disinfected with 70% ethanol and then rinsed several times in sterile water before sampling. Five to ten samples of pupal chambers and 10–15 beetles in each site per year were used for bacterial isolation. The chips of the pupal chamber and tracheae samples were infiltrated in 1 ml and 40 μl of 10% phosphate-buffered saline (PBS) separately, and the samples were sonicated for 1 min and vortexed at a medium speed for 10 s. The suspension was then plated (dilution factors varied from 10^1^ to 10^3^) on the Luria-Bertani medium (LB) and tryptic soy agar (TSA). The nystatin and cycloheximide were added for preventing the growth of fungus and yeast (Liu et al., 2021). All plates were incubated at 28°C for 15–20 days. For each morphotype, at least two cultures from different samples were selected and identified by 16S ribosomal DNA (rDNA) sequencing, and the samples were stored at −80°C in TSA broth with 20–30% glycerin.

DNA was extracted using a MightyPrep reagent for DNA (TaKaRa, Dalian, China) following the instruction. We selected 16S rDNA sequences to compare with the type strains in EzBioCloud database (https://www.ezbiocloud.net/) and combine phylogenetic relationships to identify bacteria. The 16S rDNA was amplified with primers 8F (5′-GCGGATCCGCGGCCGCTGCAGAGTTTGATCCTGGCTCAG-3′) and 1492R (5′-GGCTCGAGCGGCCGCCCGGGTTACCTTGTTACGACTT-3′) (Weisburg et al., 1991). The reaction mixture for PCR contained 12.5 μl of 2 × MightyAmp Buffer Ver.3, 0.5 μl of MightyAmp DNA Polymerase Ver.3, 1 μl of each primer (10 μM/each), and 2.5 μl of 10 × Additive for High Specificity (TaKaRa, China).

### Phylogenetic Analyses

National Center for Biotechnology Information (NCBI) and the EzBioCloud database were used to obtain sequences of the closest type strains for phylogenetic analysis (Yoon et al., 2017). A total of 118 sequences obtained from this study and 119 sequences downloaded from EzBioCloud were homologously aligned and de-tailed using MUSCLE v3.5.1 (http://www.drive5.com/muscle/) and trimAL v1.3 (http://trimal.cgenomics.org/downloads) (Edgar, 2004; Capella-Gutierrez et al., 2009). The alignment length is 1,267 bp. Finally, the phylogenetic tree (1,000 replicates) using the Maximum Likelihood (ML) method was constructed by the GTR GAMMA model in IQTree v2.1.3 (http://www.iqtree.org) (Nguyen et al., 2015; Chernomor et al., 2016). Confidence at each node was assessed by 1,000 bootstrap replicates (Zhou et al., 2021). *Anabaena affinis* (AF247591) was chosen as the outgroup. The resulting tree was visualized and edited with iTol (https://itol.embl.de) (Letunic and Bork, 2019). Bacterial sequences from pupal chambers and vector beetle tracheae used in this study have been deposited in the GenBank database under the accession numbers OM319701-OM319818.

### Nematicidal Activity of Two *Serratia* Strains

Two *Serratia* strains, *S. marcescens* strain AHPC29 and *Serratia nematodiphila* strain ZJPC33, isolated from the pupal chambers in this study were used for nematicidal activity tests. The target strain was cultured in a shaker at 28°C and 150 rpm for 24 h. After centrifuging the bacteria solution, the supernatant was removed, and the remaining bacteria pellet was resuspended in 0.9% physiological saline solution. The final bacterial concentration was diluted to 0.5 OD. The propagative stages of the PWNs used in this experiment were cultured with the fungus *Botrytis cinerea* on PDA plates at 25°C (Meng et al., 2020). Before the test, the nematodes were washed with 3% hydrogen peroxide for 10 min followed by washes with sterilized 0.03 M MgSO_4_ (Nascimento et al., 2016). After this, 200–300 nematodes were added to a 96-well sterile microplate. The ratio of nematodes to bacterial suspension was 1:1 (or control: PWN in 0.03 M MgSO_4_ solution), and the total volume was 100 ul, with five replicates per treatment. The final concentration of bacterial strains was 0.5 OD. The sterile microplates were incubated at room temperature, and nematode mortality was determined after 24 h.

### Survival Assays of Vector Beetles Exposed to Pathogenic *S. marcescens*

The two *Serratia* strains used in the nematicidal activity test were also used in survival assays of the vector beetles. The final concentration of bacterial strains for this test was consistent with the nematicidal activity test mentioned above.

The final instar larvae, early pupae (max. 2 days after pupation), and newly eclosed adults of *M. alternatus* were soaked in 70% alcohol for 30 s and then rinsed with sterile 0.9% physiological saline solution. These beetles were then added to the solution of *Serratia* bacterial cells at an OD of 0.5 in 2 ml Falcon tubes or 0.9% saline solution for control. The tubes were lightly shaken for 30 s and placed on a Petri dish (90 mm). Each treatment was replicated three times, with each replicate consisting of five beetles. Beetle survival was monitored every day for 10 days (Pineda-Castellanos et al., 2015; Raymann et al., 2018).

### Statistical Analysis

Statistical analysis was performed in R (version 3.6.1) (http://www.r-project.org) and GraphPad Prism 7. A one-way ANOVA with Tukey's multiple comparison test was used for the analysis of nematode reproduction and bacteria-to-fungi ratio, and the Kruskal-Wallis *H*-test was used for the relative abundance of the genus *Serratia* genus. The two-tailed paired *t*-test was used for the nematicidal analysis of the bacterial strains against PWN and vector beetle. The Kaplan-Meier method was used for the analysis of survival curves, and the log-rank test was applied to evaluate the significance of testing. All figures and graphs were made in GraphPad Prism 7 and adapted in Adobe Illustrator (version 23.0.5).

## Results

### Fecundity Comparison Among Sites Differing in the Duration of PWN Invasion

**Figure 1:**
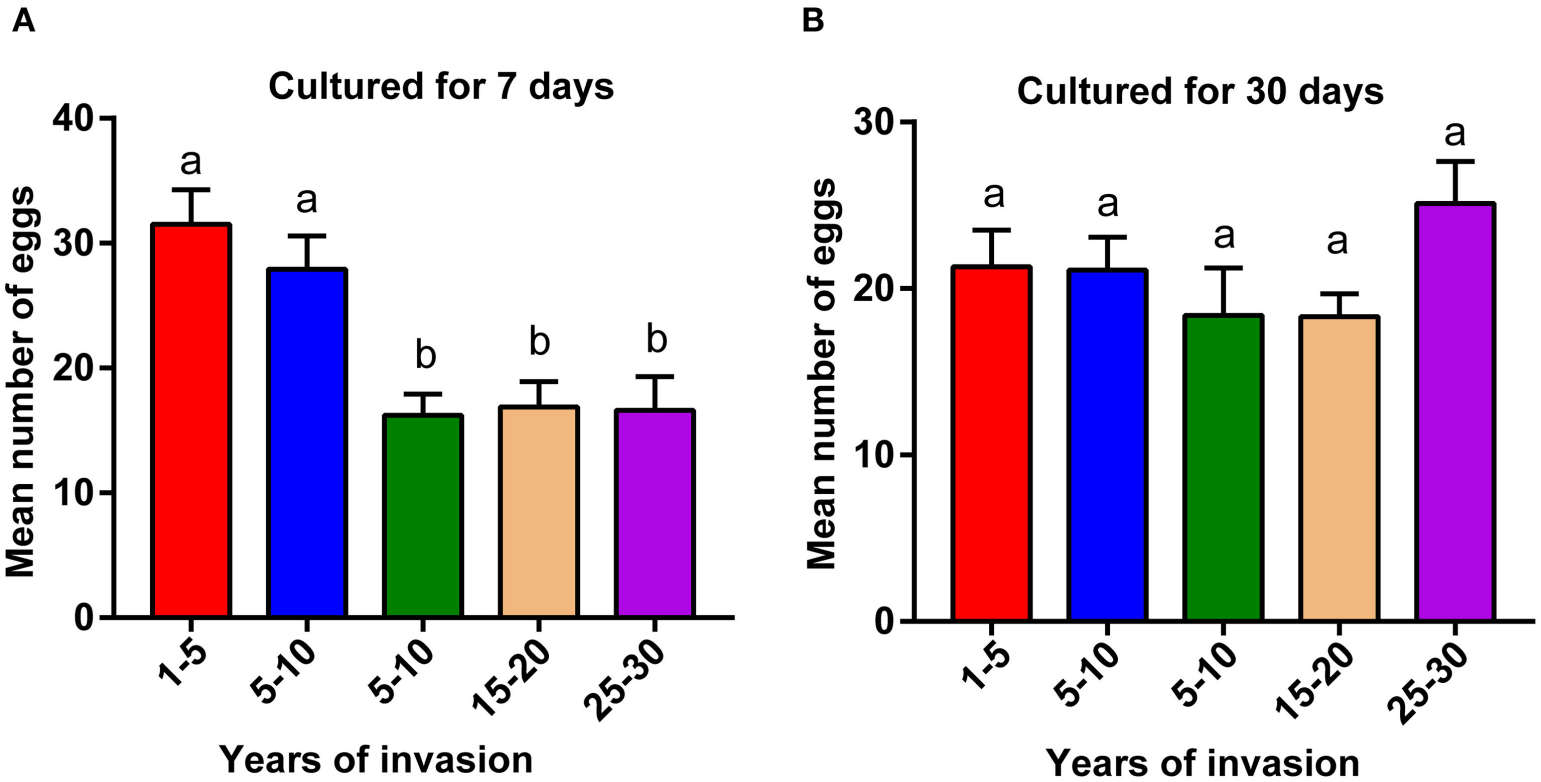
Comparison of pinewood nematode (PWN) fecundity collected from sites differing in the duration of PWN invasion. Fecundity of **(A)** wild population of PWN cultured in laboratory conditions for 7 days and **(B)** after the same populations were cultured in laboratory conditions for 30 days. A one-way ANOVA with Tukey's multiple comparison test, *p* < 0.05. Data are presented as means ± SEM. Different letters indicate significant differences. The numbers in the x-axis indicate the duration of PWN invasion in years (1–5: 1–5 years of invasion).

We compared the fecundity of PWNs collected from different sampling sites, ranging from 2 to 30 years since the PWN invasion. PWNs from sites with a longer duration of PWN invasion (>10 years) produced significantly fewer eggs compared to the PWNs collected from more recently invaded sites (<10 years) (Figure 1A, *p* < 0.05). However, after being cultured in lab conditions for 1 month, these differences in fecundity among sites disappeared (Figure 1B).

### The Bacteria-to-Fungi Ratio in Pupal Chambers and Tracheae Among Sites Differing in the Duration of PWN Invasion

**Figure 2:**
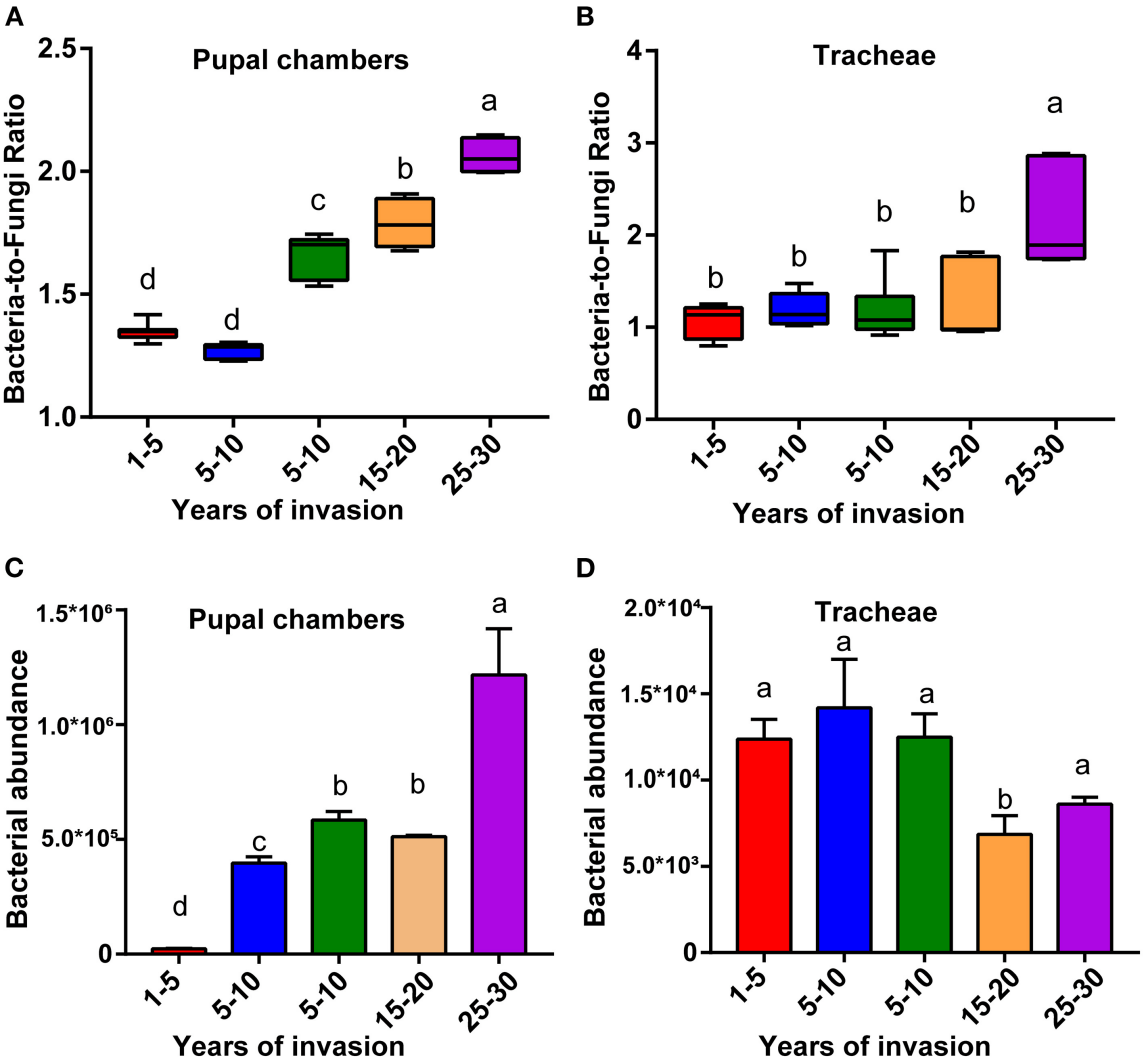
The relative bacterial and fungal abundance of pupal chambers and tracheae in sites differing in the duration of PWN invasion. The bacteria-to-fungi ratio of pupal chambers **(A)** and tracheae **(B)** samples in sites differing in the duration of PWN invasion (years of invasion in the X-axis). The total bacteria content of pupal chamber **(C)** and tracheae **(D)** samples from different sites differed in the duration of PWN Sinvasion. The abundance of bacterial and fungal biomass was estimated by RT-qPCR. Different letters indicate statistically significant differences from the one-way ANOVA with Tukey's multiple comparison test, *p* < 0.05. Data are presented as means ± SEM.

The bacteria-to-fungi ratio of the pupal chamber and tracheae samples were highest in the sites with the longest duration of invasion (25–30 years) and lowest in the sites with the shortest duration of invasion (1–5 years) (Figures 2A,B). Along with the increasing duration of PWN invasion, the bacteria-to-fungi ratio of pupal chamber and tracheae samples also gradually increased. The bacteria-to-fungi ratio in pupal chambers from the two sites with a shorter duration of invasion (<10 years) was lower than 1.5 (Figure 2A). In general, the total content of bacteria was higher compared to fungi (bacteria-to-fungi ratio >1) in all pupal chamber and tracheae samples (Figures 2A,B). The total bacteria content (estimated number of copies) in pupal chamber samples was highest in the sites with the longest duration of invasion (25–30 years), whereas the total bacteria content was lowest in sites with the shortest duration of invasion (<5 years) (Figure 2C). For the samples of vector beetle tracheae, there were no significant differences in total bacterial content among different sites, except for the site with 15–20 years of invasion, which had lower bacteria abundance compared to other sites (Figure 2D).

### Isolation and Identification of Bacterial Species

**Figure 3:**
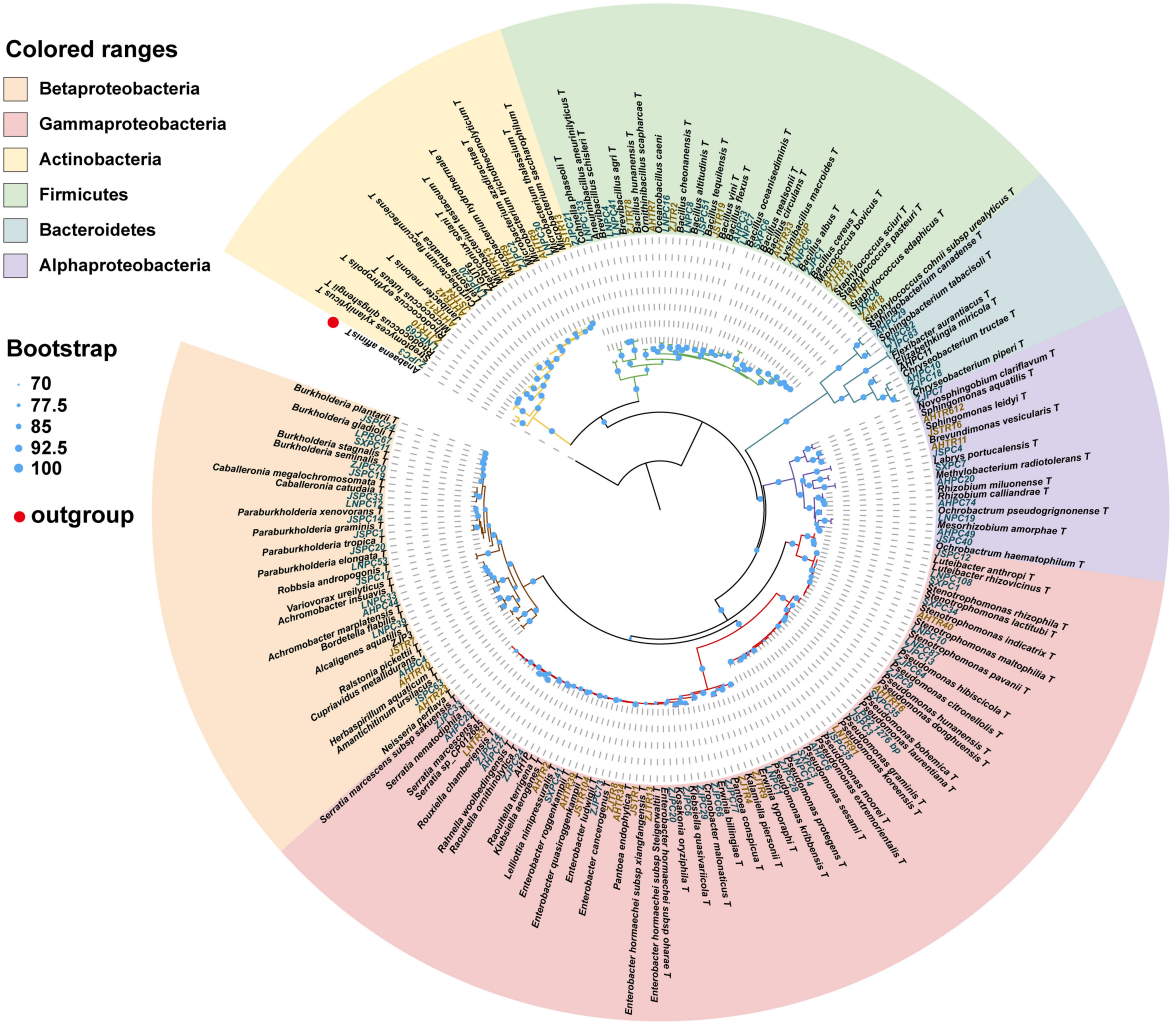
Phylogenetic tree showing the placement of 118 bacterial isolates with reference strains based on 16S rDNA sequence. The phylogenetic tree was produced by ML methods with 1,000 bootstrap replicates. Bootstrap values are represented by circles at each node. The letters in green: strains isolated from pupal chambers; letters in orange: strains isolated from tracheae. The type of strain indicated with “T”. LNPC67: bacteria strain isolated from pupal chambers in Liaoning province. AHTR10: bacteria strain isolated from tracheae in Anhui province (also see Supplementary Table 1).

We isolated and cultivated 118 bacterial strains in total, belonging to 54 genera in 30 families and from four phyla. In general, the majority of the strains belonged to the three classes of the phylum Proteobacteria, and the class of Gammaproteobacteria included six families and 15 genera (44 species). The class of Betaproteobacteria included six families and 12 genera (21 species). The class of Alphaproteobacteria included seven families and eight genera (11 species). The phylum of Actinobacteria included four families and six genera (13 species). The phylum of Firmicutes included five families and nine genera. The phylum of Bacteroidetes included two families and four genera (six species) (Table 1). A total of 28 strains belong to two or more source groups. The top five genera were *Enterobacter* (7 species), *Pseudomonas* (13 species), *Bacillus* (11 species), *Microbacterium* (6 species), and *Stenotrophomonas* (5 species) (Figure 3; Supplementary Table 1).

**Table 1:** The taxonomic distribution of bacterial isolates.

**Phylum/class**	**Family**	**Genus**	**Isolate**
Gammaproteobacteria	6	15	44
Betaproteobacteria	6	12	21
Alphaproteobacteria	7	8	11
Actinobacteria	4	6	13
Firmicutes	5	9	23
Bacteroidetes	2	4	6

This phylogenetic relationship between isolated strains and their closest type strains shown in the phylogenetic tree was also consistent with the blast result in EzBioCloud (Figure 3; Supplementary Table 1). Most phylum or class clusters strains were isolated from both pupal chamber and tracheae, except for the phylum Bacterioidetes which was only isolated from pupal chambers. The result of the phylogenetic tree showed that we isolated two species of *Serratia, S. marcescens* and *S. nematodiphila*, which had closely phylogenetic relationships (Figure 3). *Serratia marcescens* strain AHPC29 was isolated in pupal chambers and tracheae of the vector beetle from three sites with the longest duration of PWN invasion and was also found in the vector beetle tracheae in site (LN) with the shortest duration of the invasion. *S. nematodiphila* strain ZJPC33 was only isolated in the pupal chamber of ZJ in this study (Supplementary Table 1).

### The Distribution of the Genus *Serratia* Among Sites Varying in the Duration of PWN Invasion

**Figure 4:**
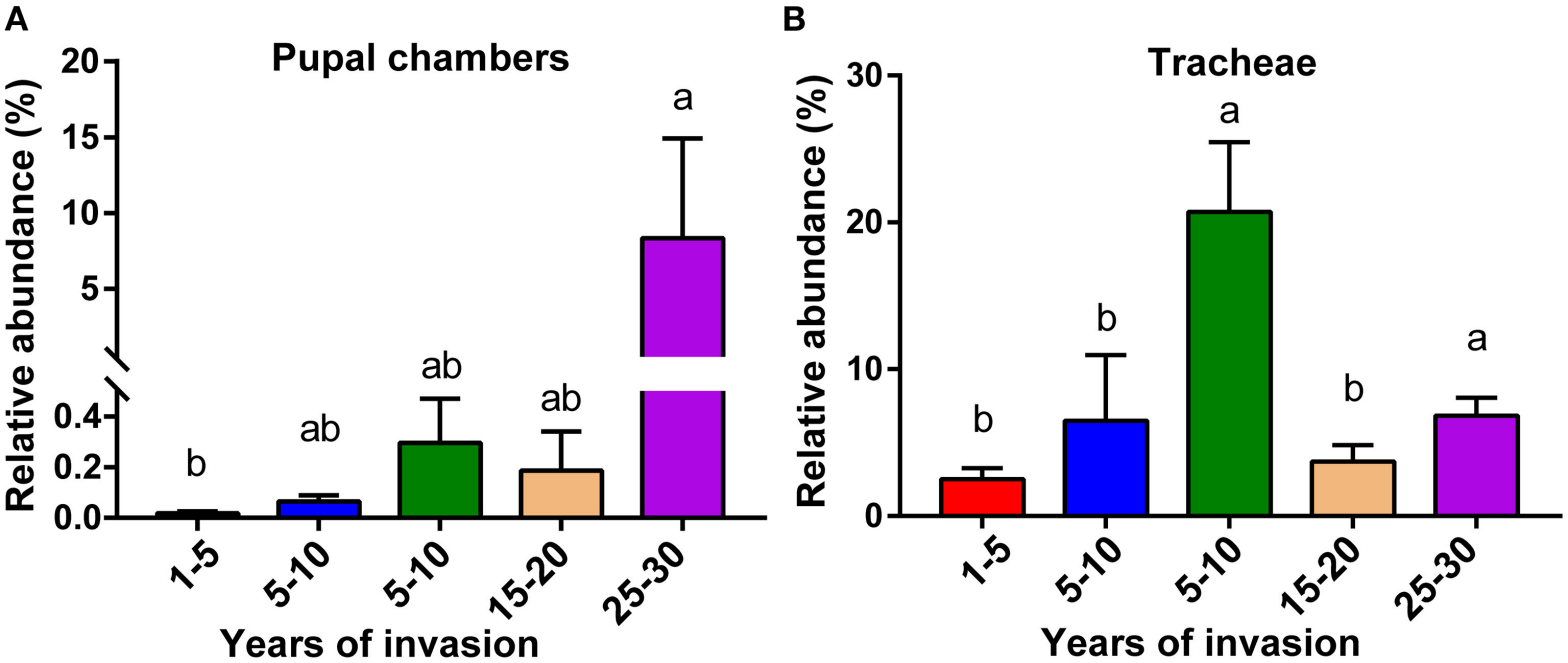
The relative abundance of genus *Serratia* involved in pupal chambers **(A)** and tracheae **(B)** from sites differing in the duration of PWN invasion (years of invasion in the X-axis). Data are presented as means ± SEM, Kruskal-Wallis *H*-test, *p* < 0.05. Different letters indicate statistically significant differences.

We analyzed the relative abundance of genus *Serratia* in the pupal chamber and vector beetle samples across the five sites differing in the duration of PWN invasion. In fact, for pupal chambers, the relative abundance of genus *Serratia* increased with the increasing duration of PWN invasion, being significantly higher in sites with the longest duration of invasion compared to sites with the shortest duration of invasion (<5 years) (Figure 4A). Only 0.093% of the genus *Serratia* was found in pupal chambers collected from sites with the shortest duration of invasion (<5 years), whereas 8.33% of this genus was detected in pupal chamber samples from areas over 20 years since invasion (Figure 4A). The relative abundance of the genus *Serratia* in vector beetle tracheae was highest in the sites with moderate (5–10 years) and the longest duration of PWN invasion (25–30 years) compared to other sites, and hence, the abundance of the genus *Serratia* did not consistently increase as the invasion progressed (Figure 4B).

### Pathogenicity of Two *Serratia* Strains to PWN and Its Vector Beetle

**Figure 5:**
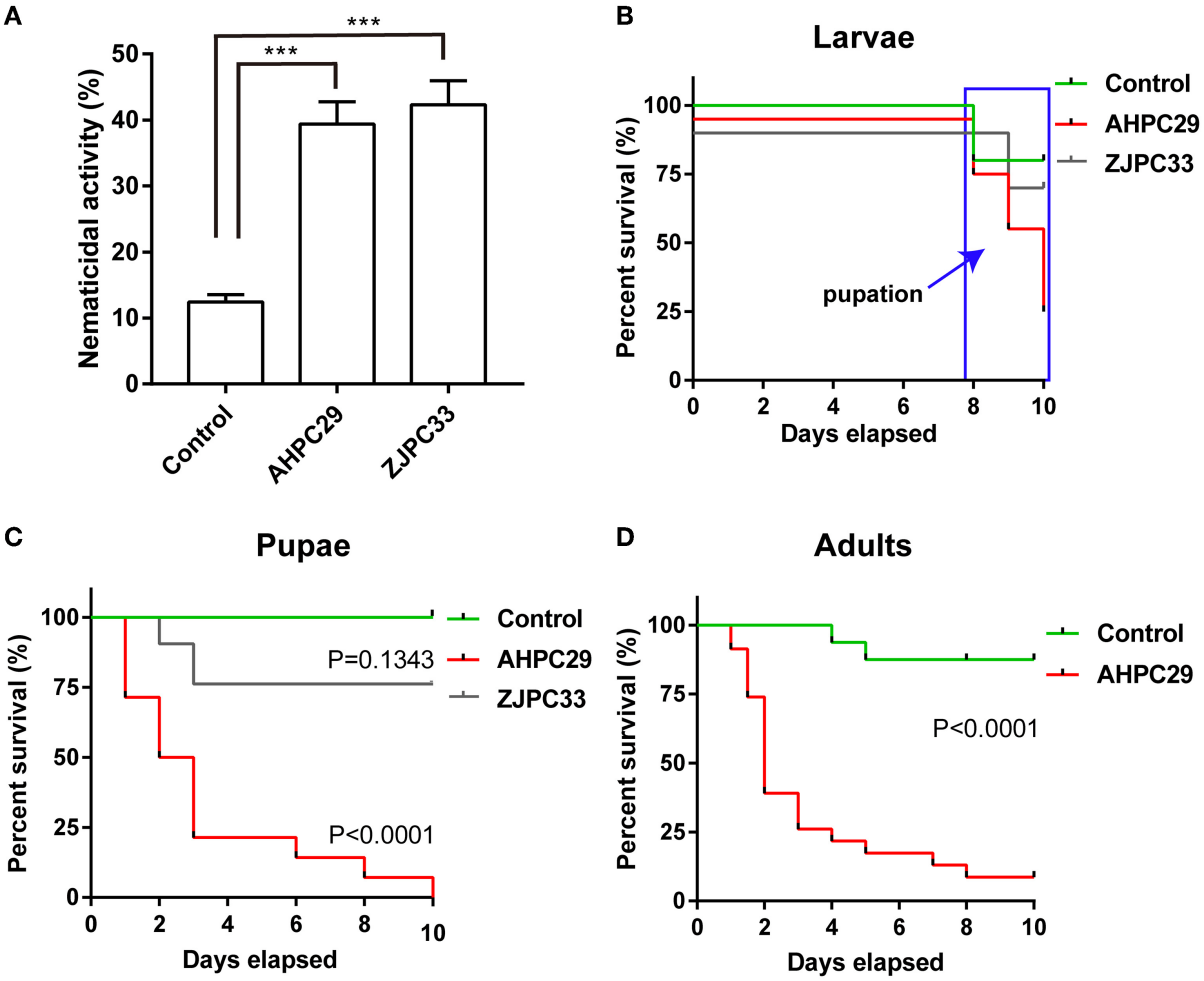
Pathogenicity of *Serratia marcescens* strain to PWN and its vector beetle. **(A)** The mortality assay of PWN with two *Serratia* strains AHPC29 and ZJPC33. AHPC29: *Serratia marcescens* strain AHPC29; ZJC33: *Serratia nematodiphila* strain ZJPC33. Nematicidal activity of bacterial solution monitored after 24 h. Two-tailed paired *t*-test, ^***^p < 0.001. **(B,C)** Meier survival curves of vector beetle last-instar larvae and pupae exposed to AHPC29 and ZJPC33. **(D)** Meier survival curves of vector beetle adults exposed to AHPC29. Survivorship was monitored daily for 10 days. The log-rank test was used for the comparison of survival curves.

We tested the nematicidal activity of the two *Serratia* species (strains AHPC29 and ZJPC33) most commonly found with pupal chambers and tracheae against PWN. The two species did not differ in nematicidal activity, *S. marcescens* strains AHPC29 caused 39.4% of mortality to PWN and *S. nematodiphila* strain ZJPC33 caused 42.3% mortality (Figure 5A).

We also tested the pathogenicity of two *Serratia* strains to vector beetle *M. alternatus*. Final-instar larvae were resistant to the AHPC29 and ZJPC33 (no mortality observed before pupation), but the resistance started to decline after pupation (Figure 5B). For the pupae stage, the ZJPC33 strain showed much lower mortality compared to the strain AHPC29 (log-rank test, AHPC29: *p* < 0.0001; ZJPC33: *p* > 0.05) (Figures 5B,C). The vector beetle adults exposed to AHPC29 showed high death rates (*p* < 0.0001; Figure 5D). Most pupae and adults died during the first 5 days post-infection of AHPC29 according to the Kaplan-Meier survival curves (log-rank test, *p* < 0.0001) (Figures 5C,D).

## Discussion

In our related previous study, we showed that the pupal chambers in sites with a longer duration of PWN invasion harbored fewer PWNs compared to sites with more recent PWN invasion (data not shown, Tian et al., Unpublished data), indicating the presence of a hidden microbial player causing negative effects to the PWN-vector beetle complex. In this study, using both quantitative detection and culture-dependent methods, we explored this possibility by investigating PWN fecundity as well as the composition and abundance of associated bacteria and fungi across five sites varying in the duration of PWN invasion (2–30 years post-invasion). PWNs collected from the sites with the longest duration of invasion had the lowest fecundity compared to PWNs collected from sites with a shorter duration of the invasion. These differences disappeared over time when the nematodes were cultured in laboratory conditions, indicating that the nematode fecundity was related to the nematode's environment within the host tree rather than site-specific genetic difference. We found indirect evidence that the reduced PWN fecundity together with the previously found lower PWN abundance may be related to the changes in the associated microbiota community and increased abundance of an antagonistic microorganism in sites with a longer duration of PWN invasion. Firstly, the bacteria-to-fungi ratio and the total bacteria content were significantly higher in sites with the longest duration of the invasion. Second, *S. marcescens*, a common bacteria found in our pupal chamber and tracheae samples, not only had the highest abundance in these sites but was also shown to be lethal to both PWN and its vector after direct exposure. Therefore, despite pupal chambers and tracheae also harboring beneficial Ophiostomatoid fungi (Zhao et al., 2013a), our results indicated that the negative effects caused by bacteria of the genus *Serratia* may override these effects and may be the “hidden players” limiting the success of the PWN-vector beetle complex when the PWN invasion has reached a certain time point.

Similar results have been demonstrated on invasive plants, showing that, as the invasion progresses over time, the initial dominance of an invasive plant species can later be reversed by stabilizing processes that are related to changes in the microbial composition (Knevel et al., 2004; Nijjer et al., 2007; Dostál et al., 2013). Soil-plant models have shown that compared to bacterial communities, fungal diversities in soil or roots are more influenced by the variation caused by the geographic location (Hu et al., 2019; Thiergart et al., 2020). Similarly, previous studies have shown that the genus *Serratia* is widespread across PWN-invaded sites (as also confirmed in the current study), whereas the dominant beneficial Ophiostomatoid fungi of the PWN-vector beetle complex are more dependent on the geographic location (Zhao et al., 2013a, 2018). This indicates that PWD-associated bacterial community may be more robust against environmental compared to fungal community and hence, may play an important role in PWD progression. However, the influence of the variation in abiotic factors, such as rainfall and temperature, among different sites may influence the abundance of bacterial and fungal associates of bark beetles (Six and Bentz, 2007; Öhrn et al., 2021), and their effects on PWD complex should also therefore be considered in future studies. Nevertheless, although not much is known about the changes in the bacterial and fungal community of invasive species in general or in terms of PWD, the increase in bacteria-to-fungi ratio in the sites with a longer duration of invasion indicates the existence of temporal change in the microbiota community in relation to the progression of PWD, and depending on the outcome of such community change, may be detrimental to the PWN-vector complex.

Similar to previous studies (Nascimento et al., 2015; Proença et al., 2017), we found that the genus *Serratia* had a strong association with both PWN and its vector beetle. The increase in the abundance of pathogenic *Serratia* bacteria in pupal chambers and tracheae in sites with a longer duration of PWN invasion, suggests that antagonistic effects against the PWN-vector beetle complex will be enhanced as PWD progresses. Besides the direct lethal effects of these bacteria on the PWN-vector beetle complex mentioned above, changes in the bacteria-to-fungi ratio may have resulted in increased microbe-microbe competition, as shown in other systems (Durán et al., 2018). This in turn may have resulted in the depletion of Ophiostomatoid fungal food source for PWN, therefore contributing to the lower abundance of PWN in sites with a longer duration of the invasion. Whether *Serratia* sp. competes with PWN-associated fungi and results in depletion of PWN's food resources or whether the lower nematode abundance and fecundity in sites with a longer duration of PWN invasion are purely caused by the nematicidal activity of such bacteria remains to be tested in future studies. The genus *Serratia* has highly diverse interactions among other organisms, depending on the interacting partner and *Serratia* species or strain. For example, although *S. marcescens* strain AHPC29 used in this study was found to be highly antagonistic to PWN and its vector, another *S. marcescens* strain associated with PWN (PWN146) has been suggested to be mutualistic, improving the virulence of PWN while being phytotoxic to pine seedlings (Vicente et al., 2012). Another *Serratia* species, *S. quinivorans* strain BXF1, is not lethal to PWN, suggesting that it may be a non-specific transient mutualist of the nematode (Nascimento et al., 2016). In addition, the genus *Serratia*, including *S. marcescens*, is also commonly found in the gut of PWNs and *Monochamus* beetles, without showing adverse effects under normal conditions (Alves et al., 2018; Guo et al., 2020; Ge et al., 2021). Considering these diverse interactions of the genus *Serratia*, the role of different species of this genus for the different players involved in PWD (host tree, PWN, and vector beetle), as well as its potential competition with the PWD associated fungi, warrant further studies. Similar to the nematicidal and insecticidal activities of *S. marcescens* demonstrated here, the pathogenicity of this species to insects has been demonstrated in other studies (Raymann et al., 2018). Several genes related to the toxicity of *Serratia* have been identified, such as those that function in lipopolysaccharide (LPS) biosynthesis, iron absorption, and hemolysin production (Hejazi and Falkiner, 1997; Kurz et al., 2003). For PWN, a kind of serine protease was found to be majorly responsible for the toxicity of the *S. marcescens* (Paiva et al., 2013), whereas, for insects, the lethal effect of this pathogen may be caused by a chitinase it produces (Hejazi and Falkiner, 1997; Lacey et al., 2015). Some studies suggest that successful colonization and pathogenicity of this bacterium may depend on the gut microbiome composition of the host (Raymann et al., 2017; Heu et al., 2021). Another mutually non-inclusive possibility is that the immune system of the inactive pupae is weaker compared to active last instar larvae, and it is also possible that *S. marcescens* is virulent only when present in the bloodstream (Raymann et al., 2018). However, because *S. marcescens* was also found to be lethal to adult beetles, further investigation is needed to confirm the mechanism behind the variation in susceptibility (e.g., immunity, cuticle thickness influencing direct exposure, and gut microbiome, among others) of different PWN vector life stages to this bacterium. Compared to previous studies investigating the nematicidal activity of *S. marcescens* using its metabolites, we used actual bacterial suspension to study its nematicidal and insecticidal activities. These differences in methodologies (different concentrations of the effective substance and/or use of different *S. marcescens* strains) may explain the relatively lower virulence observed in this study compared to previous studies, yet the virulence is within the range reported in other *Serratia* studies (Paiva et al., 2013; Proença et al., 2017; Liu et al., 2019). However, the use of live bacteria, as used in this study, is likely more ecologically relevant, more accurately mimicking the type of exposure of how the PWN and vector beetle encounter these bacteria in nature. Although the use of H_2_O_2_ to surface sterilize the nematodes resulted in some mortality in the control group, all nematodes were disinfected with this same method and hence were likely to have no significant influence on the observed mortality pattern.

The nematicidal and insecticidal *S. marcescens* strain AHPC29 used in our study could potentially be used as a biological agent against PWN and its vector beetle. One advantage of using a bacterium as a biocontrol agent is that it can more easily enter the host pine compared to chemical pesticides and is less likely to cause toxics to non-target organisms, especially because this bacterium appears to be a predominant associate of the PWN-vector complex. In addition, some studies have found that genetically engineered bacteria can improve the control of plant diseases and their insect vectors (Wang et al., 2017; Kwak et al., 2018). Therefore, the *S. marcescens* strain could also potentially be genetically engineered to carry nematicidal or insecticidal protein into host pine to control PWD. Moreover, the abundance dynamics of bacteria and fungi demonstrated in our study could reflect the prevalence of the PWN-vector beetle complex and indicate the progress of PWD. Similar to the studies showing that variation in soil and root microbiome could reflect the health status of the host plant and predict future disease outcomes (Wei et al., 2019), our results on the bacteria-to-fungi ratio could also provide important information for the evaluation of the degree of PWN invasion in field condition. Our results also found that *S. marcescens* strain AHPC29 was highly virulent for the pupae stage of vector beetle, suggesting that spring (when *Monochamus* sp. usually pupae) is likely the optimal time window for applying this control method. Future studies should investigate the pathogenicity of this strain in field conditions using different life stages of PWN and its vector beetle to confirm its applicability for PWD biocontrol.

## Conclusion

In conclusion, our results show that variation in the duration of PWN invasion can drastically alter the microbial composition and the prevalence of PWD. In addition, a common bacterial associate of PWN and its vector, *S. marcescens* (strain AHPC29), can act as an antagonistic to both of these partners required for the initiation of PWD. This lethal strain found in this study should therefore be tested in future studies (e.g., in field conditions) for its application as a biocontrol agent against PWN and its vector beetle. Our research can also indicate that microbial composition could be used as a tool to determine the progression of plant disease. For the progress of PWD, the estimate could be done by using the abundance of the genus *Serratia* as a biomarker.

## Data Availability Statement

The datasets presented in this study can be found in online repositories. The names of the repository/repositories and accession number(s) can be found below: Bacterial 16S rRNA gene sequencing data have been submitted to the NCBI BioProject under accession number PRJNA720535. Bacterial sequences from pupal chambers and vector beetle tracheae used in this study have been deposited in the GenBank database under the accession numbers OM319701-OM319818. The ASV tables used for analyses in this study are available at https://github.com/Haokai-Tian/Tian2022_microbiome.git.

## Author Contributions

HT, LZ, and JS designed the research. HT performed experiments and analyzed the data. HT, T-MK, and JS wrote the manuscript. All authors read and approved the final manuscript.

## Funding

This work was supported by the National Natural Science Foundation of China (32088102 and 32061123002) and the National Key Research and Development Program of China (2021YFC2600100).

## Conflict of Interest

The authors declare that the research was conducted in the absence of any commercial or financial relationships that could be construed as a potential conflict of interest.

## Publisher's Note

All claims expressed in this article are solely those of the authors and do not necessarily represent those of their affiliated organizations, or those of the publisher, the editors and the reviewers. Any product that may be evaluated in this article, or claim that may be made by its manufacturer, is not guaranteed or endorsed by the publisher.
