# Supplementary material for: Invasion History of the Pinewood Nematode Bursaphelenchus xylophilus Influences the Abundance of Serratia sp. in Pupal Chambers and Tracheae of Insect-Vector Monochamus alternatus
Source: Front Plant Sci. 2022 May 20;13:856841. doi: 10.3389/fpls.2022.856841 (PMC9164154; doi:10.3389/fpls.2022.856841)
Supplement: Supplementary file 1 [file Data_Sheet_1.docx]

**Supplementary Material**

**Figure S1** Map of five sites included in this study differing in the duration of PWN invasion. LN: Liaoning province; SX: Shaanxi province; AH: Anhui province; ZJ: Zhejiang province; JS: Jiangsu province. The numbers inside brackets indicate the duration of invasion in years.


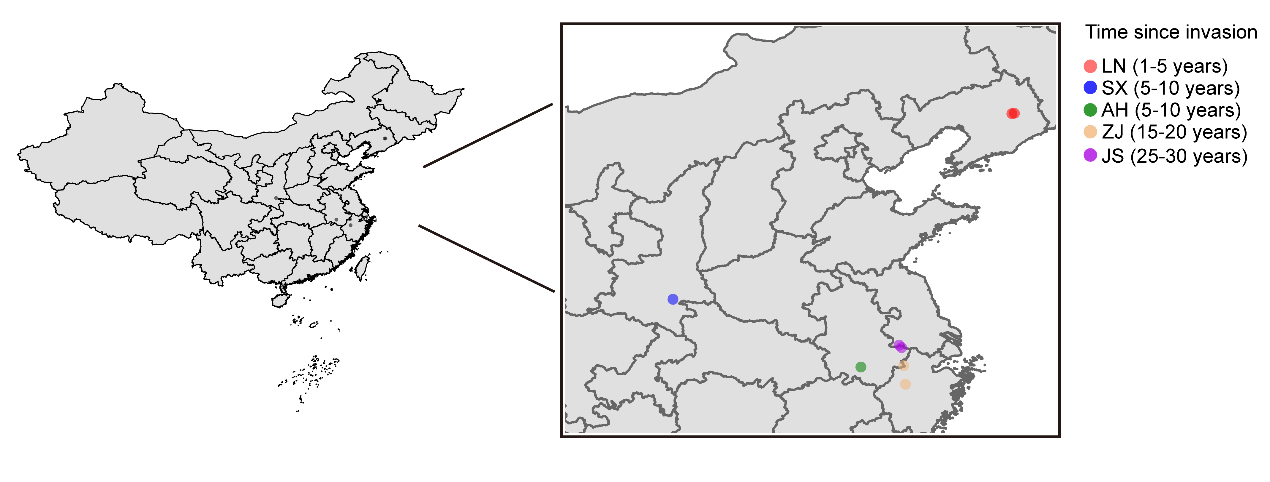


**Table S1** List of the bacterial strains isolated from pupal chambers and tracheae from five sampling sites differing the duration of PWN invasion. Bacterial strains were identified based on the 16S rDNA sequences.

| **Species affiliation** | **Closest type strains** | **Isolate numbers** | **Isolate sources** | **Similarity (%)** |
| --- | --- | --- | --- | --- |
| **Alphaproteobacteria** |  |  |  |  |
| **Brucellaceae** |  |  |  |  |
| *Ochrobactrum pseudogrignonense* | *O. pseudogrignonense* NNRM01000012 T | LNPC19 | LNPC | 99.92 |
| *Ochrobactrum teleogrylli* | *O. teleogrylli* MK063698 T | JSTR40 | JSTR/AHTR | 98.85 |
| **Caulobacteraceae** |  |  |  |  |
| *Brevundimonas vesicularis* | *B. vesicularis* BCWM01000033 T | AHTR151 | AHTR | 99.93 |
| **Labrys_f** |  |  |  |  |
| *Labrys portucalensis* | *L. portucalensis* AY362040 T | JSTR4 | JSTR | 99.64 |
| **Methylobacteriaceae** |  |  |  |  |
| *Methylobacterium radiotolerans* | *M. radiotolerans* CP001001 T | SXTR7 | SXTR | 99.57 |
| **Phyllobacteriaceae** |  |  |  |  |
| *Mesorhizobium amorphae* | *M. amorphae* AF041442 T | AHTR49 | AHTR | 99.07 |
| **Rhizobiaceae** |  |  |  |  |
| *Rhizobium calliandrae* | *R. calliandrae* JX855162 T | AHTR74 | AHTR/JSTR | 99.71 |
| *Rhizobium miluonense* | *R. miluonense* jgi.1052910 T | AHTR20 | AHTR | 99.29 |
| **Sphingomonadaceae** |  |  |  |  |
| *Novosphingobium clariflavum* | *N. clariflavum* KU530129 T | ZJPC7 | ZJPC | 98.95 |
| *Sphingomonas aquatilis* | *S. aquatilis* AF131295 T | AHTR612 | AHTR/ZJTR/JSTR | 100 |
| *Sphingomonas leidyi* | *S. leidyi* AJ227812 T | JSTR16 | JSTR/AHTR | 99.11 |
| **Betaproteobacteria** |  |  |  |  |
| **Alcaligenaceae** |  |  |  |  |
| *Achromobacter insuavis* | *A. insuavis* HF586506 T | LNPC33 | LNPC | 99.1 |
| *Achromobacter marplatensis* | *A. marplatensis* EU150134 T | AHTR44 | AHTR | 99.64 |
| *Alcaligenes aquatilis* | *A. aquatillis* JX986974 T | ZJP3 | ZJL_Ⅳ_ | 99.1 |
| *Bordetella flabilis* | *B. flabilis* CP016172 T | LNPC39 | LNPC | 98.76 |
| **Burkholderiaceae** |  |  |  |  |
| *Burkholderia gladioli* | *B. gladioli* BBJG01000151 T | LNPC67 | LNPC | 99.04 |
| *Burkholderia plantarii* | *B. plantarii* CP007212 T | JSPC24 | JSPC | 99.06 |
| *Burkholderia seminalis* | *B. seminalis* AM747631 T | ZJPC70 | ZJPC | 99.5 |
| *Burkholderia stagnalis* | *B. stagnalis* LK023502 T | SXTR11 | SXTR | 99.04 |
| *Caballeronia catudaia* | *C. catudaia* FCOF01000102 T | JSTR33 | JSTR | 98.76 |
| *Caballeronia megalochromosomata* | *C. megalochromosomata* KF155693 T | JSTR19 | JSTR | 98.76 |
| *Paraburkholderia elongata* | *P. elongata* MN723157 T | JSPC20 | JSPC | 99.09 |
| *Paraburkholderia graminis* | *P. graminis* U96939 T | JSPC14 | JSPC | 98.49 |
| *Paraburkholderia tropica* | *P. tropica* MSDZ01000065 T | JSTR1 | JSTR | 98.08 |
| *Paraburkholderia xenovorans* | *P. xenovorans* CP000270 T | LNPC12 | LNPC/LNTR/JSPC | 99.71 |
| *Robbsia andropogonis* | *R. andropogonis* LAQU01000081 T | LNPC53 | LNPC | 97.83 |
| **Comamonadaceae** |  |  |  |  |
| *Variovorax ureilyticus* | *V. ureilyticus* KU973602 T | JSPC17 | JSPC | 98.31 |
| **Neisseriaceae** |  |  |  |  |
| *Amantichitinum ursilacus* | *A. ursilacus* LAQT01000020 T | JSPC63 | JSPC | 99.71 |
| *Neisseria perflava* | *N. perflava* AJ239295 T | AHTR23 | AHTR | 99.7 |
| **Oxalobacteraceae** |  |  |  |  |
| *Herbaspirillum aquaticum* | *H. aquaticum* NJGV01000018 T | AHTR10 | AHTR | 99.5 |
| **Ralstonia_f** |  |  |  |  |
| *Cupriavidus metallidurans* | *C. metallidurans* CP000353 T | AHTR4 | AHTR/JSPC | 99.58 |
| *Ralstonia pickettii* | *R. pickettii* JOVL01000020 T | JSTR7 | JSTR/LNTR/AHTR | 99.58 |
| **Gammaproteobacteria** |  |  |  |  |
| **Enterbacteriaceae** |  |  |  |  |
| *Cronobacter malonaticus* | *C. malonaticus* AJKV01000019 T | ZJTR66 | ZJTR | 99.17 |
| *Enterobacter cancerogenus* | *E. cancerogenus* FYBA01000020 T | ZJTR8 | ZJTR | 99.64 |
| *Enterobacter hormaechei subsp. oharae* | *E. hormaechei subsp. oharae* CP017180 T | ZJTR20 | ZJTR/JSTR | 99.11 |
| *Enterobacter hormaechei subsp. Steigerwaltii* | *E. hormaechei subsp. Steigerwalti*  CP017179 T | ZJTR131 | ZJTR | 99.57 |
| *Enterobacter hormaechei subsp.Xiangfangensis* | *E. hormaechei subsp. Xiangfangensis*  FYBF01000083 T | JSTR81 | JSTR/ZJTR | 100 |
| *Enterobacter ludwigii* | *E. ludwigii* JTLO01000001 T | ZJPC73 | ZJPC | 99.5 |
| *Enterobacter quasiroggenkampii* | *E. quasiroggenkampii* KY979139 T | JSTR104 | JSTR | 99.65 |
| *Enterobacter roggenkampii* | *E. roggenkampii* CP017184 T | AHTR39 | AHTR/ZJTR | 99.5 |
| *Raoultella ornithinolytica* | *R. ornithinolytica* AJ251467 T | ZJPC24 | ZJPC | 99.65 |
| *Raoultella terrigena* | *R. terrigena* Y17658 T | AHTR1P | AHTR/AHPC/SXPC | 99.63 |
| **Erwiniaceae** |  |  |  | 99.71 |
| *Erwinia billingiae* | *E. billingiae* JN175337 T | ZJPC77 | ZJPC | 99.38 |
| *Erwinia typographi* | E. typographi GU166291 T | LNTR1 | LNTR | 99.71 |
| *Kalamiella piersonii* | *K. piersonii* RARB01000003 T | ZJTR98 | ZJTR | 98.51 |
| *Pantoea conspicua* | *P. conspicua* MLFN01000105 T | ZJTR4 | ZJTR | 99.78 |
| *Pantoea endophytica* | *P. endophytica* PJRT01000022 T | AHTR34 | AHTR | 99.79 |
| **Lysobacteraceae** |  |  |  |  |
| *Stenotrophomonas indicatrix* | *S. indicatrix* KJ452162 T | AHTR40 | AHTR | 99.5 |
| *Stenotrophomonas lactitubi* | *S. lactitubi* LT222224 T | SXPC34 | SXPC | 100 |
| *Stenotrophomonas maltophilia* | *S. maltophilia* JALV01000036 T | LNPC10 | LNPC | 99.57 |
| *Stenotrophomonas pavanii* | *Stenotrophomonas pavanii* LDJN01000038 T | LNPC87 | LNPC | 99.63 |
| *Stenotrophomonas rhizophila* | *S. rhizophila* CP007597 T | SXPC1 | SXPC | 99.72 |
| **Pseudomonadaceae** |  |  |  |  |
| *Pseudomonas bohemica* | *P. bohemica* MG190030 T | SXPC35 | SXPC/AHTR | 99.65 |
| *Pseudomonas citronellolis* | *P. citronellolis* BCZY01000096 T | ZJPC64 | ZJPC | 98.98 |
| *Pseudomonas donghuensis* | *P. donghuensis* AJJP01000212 T | AHTR16 | AHTR | 98.79 |
| *Pseudomonas extremorientalis* | *P. extremorientalis* AF405328 T | AHTR6 | AHTR | 99.18 |
| *Pseudomonas graminis* | *P. graminis* Y11150 T | JSPC3 | JSPC/ZJPC/ZJTR | 98.99 |
| *Pseudomonas hibiscicola* | *P. hibiscicola* AB021405 T | ZJTR13 | ZJTR | 99.38 |
| *Pseudomonas hunanensis* | *P. hunanensis* JX545210 T | ZJTR9 | ZJTR | 99.58 |
| *Pseudomonas koreensis* | *P. koreensis* AF468452 T | LNTR91 | LNTR/JSTR | 99.17 |
| *Pseudomonas kribbensis* | *P. kribbensis* CP029608 T | ZJPC28 | ZJPC | 100 |
| *Pseudomonas laurentiana* | *P. laurentiana*  KY471137 | LNPC2 | LNPC/SXPC/AHPC/AHTR | 99.22 |
| *Pseudomonas moorei* | *P. moorei* AM293566 T | JSPC35 | JSPC | 98.25 |
| *Pseudomonas protegens* | *P. protegens* CP003190 T | LNPC14 | LNPC/SXPC | 100 |
| *Pseudomonas sesami* | *P. sesami* EU912472 T | SXPC3 | SXPC/LNPC | 99.2 |
| **Rhodanobacteracea** |  |  |  |  |
| *Luteibacter anthropi* | *L. anthropi* FM212561 T | JSPC12 | JSPC/LNPC/ZJPC/AHTR | 98.58 |
| *Luteibacter rhizovicinus* | *L. rhizovicinus* CP017480 T | LNPC108 | LNPC/AHTR | 98.8 |
| **Yersiniaceae** |  |  |  |  |
| *Lelliottia nimipressuralis* | *L. nimipressuralis* Z96077 T | SXPC41 | SXPC | 99.35 |
| *Klebsiella aerogenes* | *K. aerogenes* CP002824 T | AHTR2 | AHTR | 98.45 |
| *Klebsiella quasivariicola* | *K. quasivariicola* CP022823 T | ZJPC29 | ZJPC | 99.57 |
| *Kosakonia oryziphila* | *K. oryziphila* JF795013T | ZJPC6 | ZJPC | 99.26 |
| *Rahnella woolbedingensis* | *R. woolbedingensis* RAHH01000065 T | AHPC27 | AHPC | 99.86 |
| *Rouxiella chamberiensis* | *R. chamberiensis* JRWU01000013 T | ZJTR14 | ZJTR | 99.18 |
| *Serratia marcescens* | *S. marcescens* JMPQ01000005 T | AHPC29 | AHPC/AHTR/ZJPC/ZJTR/JSPC/JSTR/LNTR | 99.78 |
|  |  | LNTR31 | LNTR | 99.85 |
| *Serratia nematodiphila* | *S. nematodiphila* JPUX01000001 T | ZJPC33 | ZJPC | 99.93 |
| **Actinobacteria** |  |  |  |  |
| **Intrasporangiaceae** |  |  |  |  |
| *Janibacter melonis* | *J. melonis* AY522568 T | AHTR42 | AHTR | 99.63 |
| **Microbacteriaceae** |  |  |  |  |
| *Curtobacterium flaccumfaciens* | *C. flaccumfaciens* AJ312209T | JSTR49 | JSTR/AHTR/LNPC/ZJPC | 99.56 |
| *Leifsonia aquatica* | *L. aquatica* KI271991 T | LNPC20 | LNPC/AHTR | 100 |
| *Microbacterium azadirachtae* | *M. azadirachtae* JYIT01000023 T | ZJTR2 | ZJTR | 98.05 |
| *Microbacterium hydrothermale* | *M. hydrothermale* HM222660 T | LNTR9 | LNTR | 98.46 |
| *Microbacterium saccharophilum* | *M. saccharophilum* AB736273T | JSTR13 | JSTR | 99.56 |
| *Microbacterium testaceum* | *M. testaceum* X77445 T | AHTR13 | AHTR/JSPC | 99.35 |
| *Microbacterium thalassium* | *M.thalassium* AB004713T | LNPC30 | LNPC | 98.23 |
| *Microbacterium trichothecenolyticum* | *M. trichothecenolyticum* JYJA01000006 T | AHTR9 | AHTR | 98.02 |
| *Micrococcus luteus* | *M. luteus* CP001628 T | AHTR12 | AHTR | 99.63 |
| **Nocardiaceae** |  |  |  |  |
| *Rhodococcus erythropolis* | *R. erythropolis* BCRM01000055 T | ZJTR10 | ZJTR | 99.85 |
| *Rhodococcus qingshengii* | *R. qingshengii* LRRJ01000016 T | LNPC69 | LNPC/JXPC | 99.78 |
| **Streptomycetaceae** |  |  |  |  |
| *Streptomyces xylanilyticus* | *S. xylanilyticus* LC128341 T | ZJTR3 | ZJTR | 98.55 |
| **Bacteroidetes** |  |  |  |  |
| **Flavobacteriaceae** |  |  |  |  |
| *Chryseobacterium piperi* | *C. piperi* JPRJ01000062 T | ZJPC18 | ZJPC | 97.23 |
| *Chryseobacterium tructae* | *C. tructae* FR871429 T | AHPC10 | AHPC | 98.11 |
| *Elizabethkingia miricola* | *E. miricola jgi*.1048980 T | AHTR11 | AHTR | 98.96 |
| *Flexibacter aurantiacus* | *F. aurantiacus jgi*.1107683 T | ZJPC83 | ZJPC | 99.03 |
| **Sphingobacteriaceae** |  |  |  |  |
| *Sphingobacterium canadense* | *S. canadense* AY787820 T | AHTR29 | AHTR | 98.16 |
| *Sphingobacterium tabacisoli* | *S. tabacisoli* KX129934 T | LNPC82 | LNPC | 99.93 |
| **Firmicutes** |  |  |  |  |
| **Bacillaceae** |  |  |  |  |
| *Bacillus altitudinis* | *B. altitudinis* ASJC01000029 T | LNPC8 | LNPC | 100 |
| *Bacillus cereus* | *B. cereus* AE016877 T | ZJTR61 | ZJTR | 99.25 |
| *Bacillus cheonanensis* | *B. cheonanensis* JQ966280 T | ZJTR52 | ZJTR | 99.71 |
| *Bacillus circulans* | *B. circulans* AY724690 T | AHTR33 | AHTR | 100 |
| *Bacillus flexus* | *B. flexus* BCVD01000224 T | AHTR3 | AHTR | 99.39 |
| *Bacillus hunanensis* | *B. hunanensis* HM054473 T | ZJTR78 | ZJTR | 100 |
| *Bacillus nealsonii* | *B. nealsonii* EU65611 T | SXTR6 | SXTR | 98.78 |
| *Bacillus oceanisediminis* | *B. oceanisediminis* GQ292772 T | LNTR7 | LNTR/ZJTR | 99.64 |
| *Bacillus tequilensis* | *B. tequilensis* AYTO01000043T | JSPC51 | JSPC/JXPC | 99.93 |
| *Bacillus vini* | *B. vini* KJ005123T | JSTR139 | JSTR | 99.34 |
| *Bacillus albus* | *B. albus* MAOE01000087 T | LNTR6 | LNTR | 99.59 |
| *Oceanobacillus caeni* | *O. caeni* AB275883 T | LNPC16 | LNPC | 99.78 |
| *Ornithinibacillus scapharcae* | *O. scapharcae* AEWH01000025 T | AHTR7 | AHTR | 98.58 |
| **Paenibacillaceae** |  |  |  |  |
| *Aneurinibacillus aneurinilyticus* | *A. aneurinilyticus* KE952670 T | LNPC133 | LNPC/LNTR | 99.93 |
| *Brevibacillus agri* | *B. agri* D78454 T | LNPC41 | LNPC/LNTR/ZJTR | 99.41 |
| *Brevibacillus schisleri* | *B. schisleri* MXAR01000082 T | LNTR4 | LNTR | 98.93 |
| *Cohnella phaseoli* | *C. phaseoli* EU014872 T | ZJTR21 | ZJTR | 98.97 |
| **Planococcaceae** |  |  |  |  |
| *Lysinibacillus macroides* | *L. macroides* LGCI01000008 T | AHTR40P | AHTR | 99.18 |
| **Staphylococcaceae** |  |  |  |  |
| *Macrococcus bovicus* | *M. bovicus* Y15714 T | AHTR8 | AHTR | 100 |
| *Staphylococcus edaphicus* | *S. edaphicus* KY315825 T | JXPC8 | JXPC | 99.31 |
| *Staphylococcus pasteuri* | *S. pasteuri* AF041361 T | LNTR11 | LNTR | 99.22 |
| *Staphylococcus sciuri* | *S. sciuri* AJ421446 T | ZJTR12 | ZJTR | 99.37 |
| *Staphylococcus ureilyticus* | *S. urealyticus* AB009936 T | ZJTR18 | ZJTR | 99.93 |
|  |  |  |  |  |
|  |  |  |  |  |
